# Children born to women in opioid maintenance treatment: A longitudinal study of child behavioral problems and parenting stress

**DOI:** 10.3389/fped.2022.1087956

**Published:** 2022-12-23

**Authors:** Monica Sarfi, Marie Eikemo, Carolien Konijnenberg

**Affiliations:** ^1^ Norwegian Centre for Addiction Research (SERAF), Faculty of Medicine, University of Oslo, Oslo, Norway; ^2^ Department of Psychology, Faculty of Social Sciences, University of Oslo, Oslo, Norway; ^3^ Department of Psychology, Inland Norway University of Applied Sciences, Lillehammer, Norway

**Keywords:** prenatal opioid exposure, development, methadone, buprenorphine, parenting distress

## Abstract

In the wake of the “opioid epidemic”, there is considerable concern regarding potential harmful long-term effects of prenatal opioid exposure. Opioid misuse and addiction confer increased exposure to lifestyle stressors and health burdens. Accordingly, it is challenging to disentangle effects of prenatal opioid exposure *per se* from factors related to maternal stress. In this study, we followed 36 women enrolled in comprehensive opioid maintenance treatment (OMT) program and their children alongside 36 age-matched mother-child dyads from a community sample (COMP) from pregnancy until child-age 8 years. Across five sessions, we used a battery of well-established questionnaires to investigate trajectories of parenting stress and mental health symptoms as well as child behavior problems. The 8-year retention was relatively high (OMT: 72%, COMP: 67%), and the OMT sample remarkably stable and well-functioning, with minimal concomitant illicit drug use. Mixed effects regressions showed significantly different trajectories of child behavior problems (*F* = 3.8, *p* = 0.024) and parenting stress (*F* = 3.1, *p* = 0.016) in the two groups. Differences in experienced stress were largely explained by more distress specifically related to the parenting role in the OMT group (*F* = 9.7, *p* = 0.003). The OMT sample also reported higher psychological distress (*F* = 15.6, *p* < 0.001) than the comparison group, but notably few participants presented with problems that warranted clinical intervention. The results underscore the benefits of tailored follow-up of children prenatally exposed to opioids and their families beyond infancy and toddlerhood. Long-term direct effects of prenatal opioid exposure on behavior problems are likely modest, given an otherwise stable caregiving environment conducive to healthy development.

## 1. Introduction

The increased access to both licit and illicit opioids globally has received pronounced public health and scientific attention as it has affected the lives of millions of individuals. Of these, many are parents or child caregivers. Opioid dependence, and the resulting impact on parental capacity raises major concerns regarding the well-being and safety of the children in these households (1, 2). Opioid maintenance treatment (OMT) with methadone or buprenorphine is an established and recommended “best practice” intervention for pregnant women with opioid use disorders (OUD) (3). Like illicit opioids, methadone and buprenorphine mimic naturally occurring endorphins and activate the same opioid receptors. However, they do so more slowly than other opioids, thereby preventing erratic maternal opioid levels and protecting the fetus from repeated episodes of withdrawal (4). OMT is associated with healthier pregnancies, lower risk for miscarriage, better access to prenatal care for the woman and significantly improved birth outcomes compared to untreated opioid use disorder (5) and tapering in pregnancy (6). Stability in OMT has shown to reduce reported lifestyle problems and stress associated with illicit drug use (7) and has improved the quality of the home environment for children of parents in OMT. Despite the beneficial effects, concerns are often raised regarding the possible negative consequences of prenatal exposure to OMT medications on the developmental outcomes of the children.

Prenatal exposure to any opioid agonist has an immediate effect on the newborn, often resulting in neonatal abstinence syndrome (NAS), indicating opioid withdrawal. While symptoms of NAS often abate within days or weeks, there is lacking consensus regarding possible harmful long-term consequences on developmental outcomes and studies show varying results, depending on methodology and outcome measures (8, 9). However, results from a rigorous longitudinal randomized controlled trial showed that children exposed to opioid agonists prenatally follow a pattern of normal development during the first 3 years of life (10).

Studies of effects of prenatal opioid exposure beyond 3 years are few and show divergent findings (11). Some reports indicate that opioid-exposed children have higher risk of difficulties in childhood such as cognitive, neuro—and psychomotor development (12), mediated in part through suboptimal maternal caregiving. Other studies show heightened internalizing and externalizing behavior problems, conduct disorders and ADHD diagnoses (13). However, there is little evidence to support direct relationships between prenatal opioid exposure and adverse developmental trajectories into later stages of childhood and adolescence. Rather, the adverse effects observed on child outcomes appear to be mediated and moderated by a number of individual and environmental factors and the interplay between these factors, not the opioid exposure *per se* (11).

Addiction treatment alone may not be sufficient to address the underlying factors that can affect child safety and development in families with OUD. This vision is embedded in the Norwegian OMT program, which aims to provide comprehensive, collaborative care for opioid-addicted parents and their children within the framework of the free national public health care system. Pregnant patients enrolled in the national OMT program in this country are subjected to strict control routines such as regular urine tests, counselling, regular pregnancy checkups and child welfare referral when required. These national guidelines for pregnant OMT patients and their children outline a structured follow-up regimen from birth to school-age (14), involving hospital services as well as a range of health professionals in the field of addiction treatment, mental health and education services. As a result, studies on OMT cohorts in this country have shown very little concomitant drug use in pregnancy and birth parameters of children born to mothers in OMT are within the normal ranges (15, 16). A substantial number of mothers retain custody 8 years after delivery and a recent study showed that children growing up with parents in stable OMT have significantly better mental health in early school age than other vulnerable groups such as children placed in foster care (17).

Despite good retention and rehabilitation, mothers in OMT often share many of the difficulties of addicted mothers outside OMT such as socioeconomic and interpersonal challenges. There is also a prominent fear of losing custody of children in this group of mothers, which is dependent on adherence to the schedules and rules in the OMT program. On top of contextual risk factors, women with OUD have high risk for comorbid psychopathology—in particular mood disturbances—that influence child care which in turn may account for reduced distress tolerance in the parenting role (18). At the neurobiological level, caregiving challenges observed in parents with opioid addiction may reflect the general dysregulation of neural circuits underpinning reward and stress responses seen in addiction (19), which are also important for parenting (20, 21).

Parenting stress is one of the most prominent sources of stress and is experienced by all parents to some degree (22–24). Parenting stress accounts for the stress associated specifically with the parenting role and is influenced by factors residing both within each parent and factors in their environments (25, 26). There is compelling evidence that mothers with opioid addiction typically have more stress in their lives compared to mothers from normative samples (27). Less is known about how OUD treatment and treatment stability enable opioid-addicted mothers to manage parenting over time,—especially faced with personal and child related challenges. Studies of early mother-child interaction have consistently found patterns of poor sensitivity and responsiveness to infants’ emotional and behavioral cues in dyads of substance-dependent mothers compared to normative dyads (28, 29). However, difficulties with sensitive parenting are multiply determined and may be compounded by infants’ display of “difficult behaviors” such as fussiness and disrupted sleep pattern—often occurring in infants exposed to opioids *in utero* and known as neonatal abstinence syndrome (NAS) (30). Parenting an infant with regulatory problems or raising a child with behavioral challenges tends to increase parenting stress, and parents who experience greater parenting-related stress may be more likely to parent in ways that maintain child problems or even put the child at risk for maltreatment (31, 32).

While many studies have assessed parenting in substance using populations, outcomes reported are frequently confounded by factors as parental concomitant substance use and psychiatric comorbidity in the study groups. As such, longitudinal studies of children of mothers in stable OMT are needed to explore developmental consequences of prenatal opioid exposure *per se*.

The longitudinal data presented here stem from a prospective observational study of mothers enrolled in OMT in pregnancy and their children exposed to opioid agonists prenatally who were raised in relatively stable home environments. In parallel, an age matched comparison group of non-exposed children and mothers with no history of drug addiction was followed. Participants in the OMT study group had been in this treatment on average 2.5 years at study inclusion and were stable in treatment throughout the study period. Further, this is a group with very limited concomitant substance use and less psychological distress symptoms than reported in similar study groups, i.e., (33). Also, 80% of the included children lived with biological parents when they were 8 years. Both study cohorts were followed from pregnancy to school-age.

Specifically, we aimed to describe (a) parenting stress, (b) perceived child behavioral problems, and (c) post-natal mental health of mothers in OMT and a comparison group of non-dependent mothers from infancy to early school age and to explore the association between child behavior problems and parenting stress in the two groups across time.

## 2. Methods and materials

To date, OMT in Norway includes ∼8,000 individuals, one-third being women. The number of pregnancies in the OMT program has been low and stable in the period 2005–2015 with a mean number of 28 pregnancies per year, representing 0.06% of the general pregnant population in Norway during the same time period (34).

### 2.1. Participants

#### 2.1.1. Participants in opioid maintenance treatment

Data included in this study is part of a prospective, longitudinal cohort study of children born to mothers in opioid maintenance treatment in Norway who were included during a 2-year period (2005–07). Around 30% of the OMT population of 7,500 individuals in the country were women. The annual birth rate of children exposed to OMT-medications has varied between 25 and 40 since OMT was introduced as treatment option in 1998.

Women in the OMT-group were contacted if they had a pregnancy due date between January 2005 and January 2007 and had used either methadone or buprenorphine during pregnancy. Recruitment took place through local GP contacts, regional OMT centers and treatment facilities throughout Norway. Of the 47 pregnant women in OMT in Norway identified at the start of the longitudinal project, six declined to be included, two miscarried, one child was excluded due to a severe congenital disorder which yielded a 76% participation rate (*N* = 36). A majority of the mothers (68%) in OMT had been in stable in this treatment for a considerable time before pregnancy was confirmed (31.1 months, range: 3–81 months) and used methadone as their OMT-medication while the rest used buprenorphine. There was very little concomitant illicit drug use (35), but almost all mothers in the OMT group smoked cigarettes daily during pregnancy.

#### 2.1.2. The community sample comparison group

Describing the trajectories of parenting stress and child behavior problems *within* the OMT group was the main goal of this study. It was not feasible to recruit a control group matched on socio-economic/demographic variables for a long-term follow-up study in Norway, due to high living standard and free healthcare and social services. Nevertheless, an age matched community sample of healthy pregnant women without illicit drug use or psychiatric illness were recruited to serve as a “comparison group” (COMP, *N* = 36) as a means to address general developmental trajectories throughout the follow-up period. The COMP participants were recruited through local health care centers in and around the capital city. Importantly, potential main effects of group may therefore be confounded by socioeconomic and opioid-related influences. On the other hand, an absence of overall group effects may suggest a rather well-functioning OMT group. Accordingly, we are primarily interested in age*group interaction effects that could indicate differences in developmental trajectories. Information about the infants (i.e., weight, presence of neonatal abstinence symptoms) was obtained directly from hospital medical records. Recruitment and inclusion procedures are described in more detail in previous publications (36, 37).

Descriptive statistics and socio-demographic variables are in the two groups at study inclusion and 8 years later are shown in Table 1. The proportion of women in OMT in employment or education-related activities increased during the study period, and more disclosed having a stable partner. The rate of smoking was unchanged and high in the OMT group.

**Table 1:** Key characteristics of the two study groups at delivery and 8 years later.

Variables	Birth		8 years	
	OMT (*n* = 36)	COMP (*n* = 36)	OMT (*n* = 26)	COMP (*n* = 24)
NAS yes/no	23/13 (63.9%)	–	–	–
Birthweight (g) *M* (SD)	3,146 (599)	3,618 (343)	–	–
Age mother *M* (SD)	32.2 (4.7)	32.6 (4.7)	40.0 (5.0)	40.0 (4.7)
Gestational age *M* (SD)	38.6 (2.5)	40.0 (0.7)	–	–
Methadone *N*	26	–	15	–
Dose mg, *M* (range)	108.5 (0–660)	–	120 (0–440)	–
Buprenorphine, *N*	10	–	11	–
Dose mg, *M* (range)	13.3 (3–24)	–	12.0 (4–20)	–
Smoking yes/no	35/1	0/36	22/4	3/21^a^
Work or study yes/no	3/33	35/1	10/16	24/0
Partner yes/no	11/25	36/0	18/8	23/1

Methadone and buprenorphine doses are not comparable because some of the women converted medication during the study period. All participants were white, Norwegian women.

^a^ Women in the comparison group who reported smoking were party smokers.

**Figure 1:**
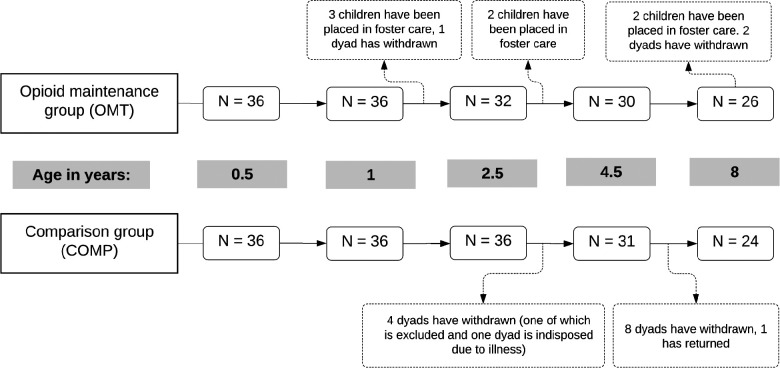
Schematic of retention and discontinuation in the two groups across the five assessment points. Age refers to child age in years.

Some of the children in the OMT group were placed out-of-home during study period, and foster-parent reported information was collected after placement. Nevertheless, only information given by the biological mothers (original mother-child dyads) at each assessment point was used in the present study. All available data from these mothers was used, even those with missing data for some assessments. The total number of participants with complete data sets amounted to 26 mothers in the OMT group and 24 comparison mothers (see Figure 1).

### 2.2. Procedures

Participants received verbal and written information about the study at the time of recruitment and signed consent forms with permission to be contacted again for further assessments as the child grew older. They also signed separate consent forms prior to each assessment. Data was collected in pregnancy, during infancy (3 and 6 months), in toddlerhood (1 and 2.5 years), preschool period (4.5 years), and school age (8 years). The study was approved by the Regional Ethics Committee (2013/1606/REK Sør-Øst B) and conducted in accordance with the Declaration of Helsinki (1964).

Previous publications from this study group have addressed various aspects of both parental and child functioning (17, 35, 38–40) throughout the 8-year follow-up-period. Here we analyze key outcomes across the whole study period, focusing primarily on parenting stress and child behavioral problems.

#### 2.2.1. Attrition and retention rates

During the 8-year study period, five boys and two girls in the OMT group were placed out-of-home by Child Welfare Services. Three children were removed from home between the 1 and 2-year assessments, and one mother withdrew from the study shortly after the child was 1 year old (see Figure 1). Two children were placed in foster care before the 4-year assessment, and another two children before the 8-year assessment. The two mothers who discontinued the study prior to the 8-year assessment did so because they feared potential stigma. The COMP group was reduced to 31 participants at the 4-year assessment (85%) because four families withdrew from the study, one mother was temporarily indisposed by illness (but returned at the next assessment point) and one other child from this group was excluded prior to the third assessment due to cerebral palsy. The number of participants in the COMP group was further decreased at the last assessment as 8 more mothers withdrew from the study. All the mothers in the COMP group who discontinued stated time constraints as their main reason to leave the study.

### 2.3. Measures

#### 2.3.1. The parenting stress index (PSI)

The PSI is a self-report questionnaire specifically developed to identify potential child and parent characteristics that might lead to stress in the parenting system (41, 42). The original PSI consists of 120-items scored on a five-point Likert type scale, with responses ranging from strongly agree (1) to strongly disagree (5). Higher scores indicate higher stress. The PSI has a Parent and Child Domain that in sum reflects the overall degree of stress in the parenting system. An abbreviated version of the PSI was developed in 1990 and is referred to the Short form version (PSI-SF) (41). The PSI-SF is a direct derivative of the full-length version and takes approximately 10 min to complete. It consists of 36 items built upon Castaldi's 1990 factor analysis of the original (here referred to as the long form: PSI-LF) which demonstrated that the parenting stress construct consisted of three central factors. The PSI-SF consists of three subscales namely *Parental Distress, PD* (“I feel trapped by my responsibilities as a parent”; “I feel lonely and without friends”), *Parent-Child Dysfunctional Interaction, PCDI* (“Sometimes I feel my child doesn't like me and doesn't want to be close to me”, “When I do things for my child, I get the feeling that my efforts are not appreciated”). *Difficult Child, DC* (“My child makes more demands on me than most children”, “My child gets upset easily over the smallest thing”).

Each subscale consists of 12 items in statement form. Agreement is rated from 1 (strongly disagree) to 5 (strongly agree), with subscales scores ranging from 12 to 60. A total PSI-SF score is calculated by summing the three subscales’ scores, ranging from 36 to 180. Scores above the 85th percentile on the Total Stress scale are considered borderline clinically significant Similarly, the cut-off scores for the subscales are 33 (PD), 26 (P-CDI) and 33 (DC) (43).

The two versions of the PSI have been in use at different time points in the present study due to time constraints. The PSI-SF was completed by parents at the first and last assessment (6 months and 8 years). The full-length PSI was administered at age 1, 2.5 and 4.5. The PSI-SF is a direct derivative of the full-length version and has been used in many studies (44). Here we converted all data to short form to formally model parenting stress over time. The 36 equal-wording items that constitute the PSI-SF were extracted from the full-length versions and plotted into PSI-SF templates. The scale reliability of the PSI-LF and the PSI-SF supports this construction procedure as shown by good inter-item reliability (Cronbach's alpha) for the original PSI-LF and PSI-SF as well as the three constructed PSI-SF questionnaires (see Table 2).

**Table 2:** Cronbach's alpha for the different PSI versions.

	PSI Version	
Age (years)	PSI_LF	PSI_SF
0.5	NA	0.9
1.0	0.94	0.88^a^
2.5	0.93	0.83^a^
4.5	0.94	0.91^a^
8.0	NA	0.94

Cronbach's alpha.

^a^ Denotes the values computed for short form versions constructed from items in the long form of the PSI.

#### 2.3.2. The Edinburgh postnatal depression scale (EPDS)

The EPDS is a set of ten screening questions that can indicate whether a parent has symptoms that are common in women with depression and anxiety during pregnancy and in the year following the birth of a child (45). To complete this set of questions, the parent should select the number next to the response that comes closest to how they have felt in the past 7 days. Responses are scored 0, 1, 2 and 3 based on the seriousness of the symptom). The total score is found by adding together the scores for each of the 10 items. Based on a number of studies, a cut-off of 13 or higher could be used to identify pregnant and postpartum women with higher symptom levels, whereas lower cut-off values could be used if the intention is to avoid false negatives and identify most patients who meet diagnostic criteria (46).

#### 2.3.3. The hopkins symptom checklist-25 (SCL-25)

The SCL-25 is a widely used screening tool for measuring anxiety and depression in both clinical and normative samples (SCL-25, 47). It comprises a 10-item subscale for anxiety and a 15-item subscale for depression. In the version used here, each item relating to a symptom is rated from 0 (none) to 4 (very much). Scores for each subscale were computed as averages across the 15 depression items and 10 anxiety items. In accordance with a previous study using this questionnaire version, a cut-off of ≥1.0 was used to identify participants with at least some distress (48). In the present study, SCL-25 data was available from measurements in pregnancy, 6 months after delivery and at child ages 2.5 and 8 years.

#### 2.3.4. Child behavior checklist (CBCL 1 ½-5)

This 100-item checklist (49) measures specific emotional and behavioral problems of children ages 18 months through 5 years. The questionnaire is administered to parents or other caregivers who know the child well. Caregivers rate items describing statements relating to behavior on a scale from zero to two, with higher scores indicating greater problem severity. Items are summed to make up seven subscales (emotionally reactive, anxious/depressed, somatic complaints, withdrawn, sleep problems, attention problems and aggressive behavior) which in turn can be combined into two higher-order scales; internalizing and externalizing problems, and a total difficulty score. The minimum possible score is 0 and the maximum is 200. In this study, the Child Behavior Checklist (CBCL 1 ½-5) was administered to mothers at child ages 2.5 and 4 years.

#### 2.3.5. Strengths and difficulties questionnaire (SDQ)

The parent version of the SDQ is designed for children aged 4–16 years. The questionnaire consists of 25 items distributed on 5 subscales of five items each (emotional problems, conduct problems, hyperactivity/inattention problems, peer problems and prosocial behavior). The first four scales are summed to calculate a *total difficulties score* (0–40), used here. Higher scores one the SDQ indicate more difficulties. This questionnaire was completed by mothers at the last assessments (8 years).

##### 2.3.5.1. Common scale for behavior scores

The CBCL and the SDQ are used to measure the same underlying construct: behavioral difficulties. The number of items and composite scales differ. However, previous studies show that the sum scores from the two instruments are strongly correlated (50, 51). To enable longitudinal analysis across time points, we chose to rescale both scores to 0–1. Ratings were converted to a number between 0 and 1, based on the minimal and maximal possible score in each instrument such that new score would reflect the “relative problem load”. Here we used the new score for inferential statistics, but also report raw scores from each questionnaire (total scores). Sensitivity analyses of main group differences were conducted to assess the face validity of the common scale score (see Supplementary Material).

### 2.4. Statistical analysis

All analyses were performed using R version 4.1.0 (packages are described in the Supplementary Material). For PSI, child behavior scores and SCL data, mixed effects regressions were used to account for dependencies in the data. Models were implemented in the *lme4* package in R and assessments nested within mother (subject). Mixed effects models allow easy inclusion of covariates at within- and between subject levels as well as different random effect variables. Mixed models are also flexible with regards to unequal group size and some types of missing data. A random intercept for participant was used in all models to account for the non-independence of data within participant. Age was modeled as a categorical predictor due to the limited number of assessments and relative variability in the measurement times. All models included the design relevant fixed effects *group* and *age* and *group*-by-*age* interaction. Variance explained by inter-individual differences in *birthweight* and *sex*, (and *years in treatment)* were tested during model selection for the analyses of PSI scores and problem behavior. A decrease in Bayesian Information Criterion (BIC) of 2 or more was used as an indication of a superior model. For the final models, the results from REML model are reported. Overall contrasts were performed with Satterthwaite's method for denominator degrees of freedom. Tukey correction for multiple comparisons were used for *post hoc* contrasts. A separate regression was performed on the OMT data to assess whether neonatal abstinence syndrome (NAS) explained variance in the reports of behavior difficulties or stress. Group differences in the EDPS data were assessed with an independent samples Welch's *t*-test, which is robust to unequal variances, was used to test for group differences in the postnatal depression (EDPS) data. A significance level of 5% was used for all analyses. Internal consistency of the converted PSI scales was assessed by Cronbach alpha (*α*). We also report Pearson correlations to describe the associations between the two main outcomes (total PSI score and child behavior scores) at the three time points where both measures were collected (2.5, 4.5 and 8 years).

A third of the participants discontinued or were excluded during the follow-up period (*n* = 22). To assess whether the participants who discontinued had worse mental health at the first assessment (in pregnancy) compared to those that completed the whole study, we conducted a sensitivity analysis comparing SCL-25 scores at the first assessment (Welch's *t*, *completed* vs. *discontinued*).

## 3. Results

### 3.1. Reported parenting stress

#### 3.1.1. Total parenting stress

**Figure 2:**
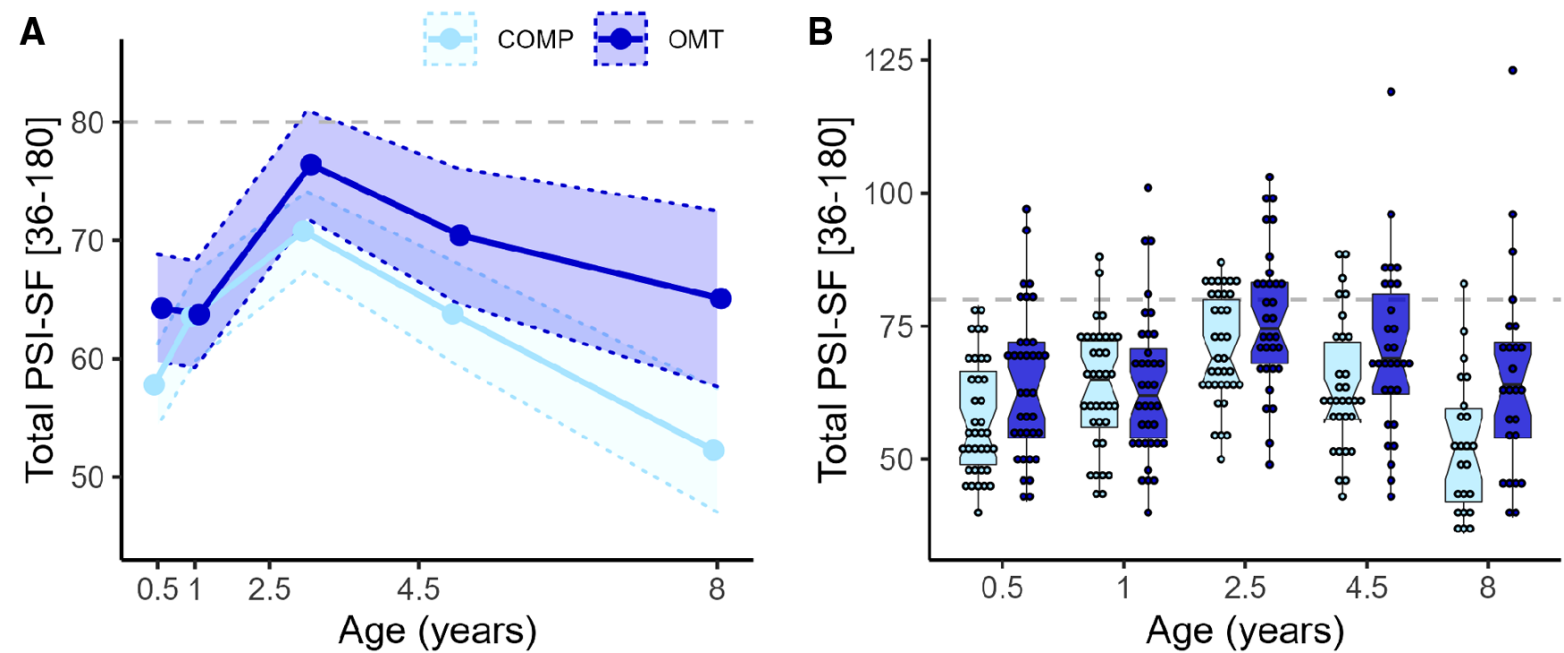
Showing the trend in PSI over time and scores for each participant (**A**) group-wise mean total PSI-SF with 95% confidence bands for the five time points. Age is presented on numerical scale on the x-axis to illustrate relative timing of assessments. (**B**) Box- and dot plots of total PSI-SF scores presented group-wise at each time point. OMT: dark blue, COMP: light blue. The box plot notches represent the 95% CI of the median in the middle line). Dots show the total PSI-SF score for each participant and each point of assessment, presented on a categorical x-axis. Dashed grey lines indicate the 80-point threshold, often used as a cut-off for clinical problems.

Figure 2 shows the distribution of parenting stress scores at each assessment. Total PSI-SF scores were on average 7 points higher in the OMT group (*M*_OMT_ = 69, *M*_COMP_ = 62). The mixed regression for the total PSI score showed a significant main effect of *group* (*F*_1,74.1 _= 5.5, *p* = 0.022) and *age* (*F*_4,253.5 _= 24.5, *p* < 0.0001) and age*group interaction (*F*_4,253.5 _= 3.1, *p* = 0.016). Pairwise comparisons showed significant group differences at the first (6 months: Mean_Diff_ OMT > COMP = 6.5, *p* = 0.038) and last assessment (8 years, OMT > COMP 11.9, *p* = 0.0008). A separate regression of PSI in the OMT group only, showed that neither *time in OMT treatment* nor neonatal abstinence syndrome (*NAS*) were significant predictors of parenting stress, and did not improve model fit. The intraclass correlation (ICC) was 0.52, indicating relatively high consistency in reports across assessments within-subject.

##### 3.1.1.1. Sub-scales of the PSI

**Figure 3:**
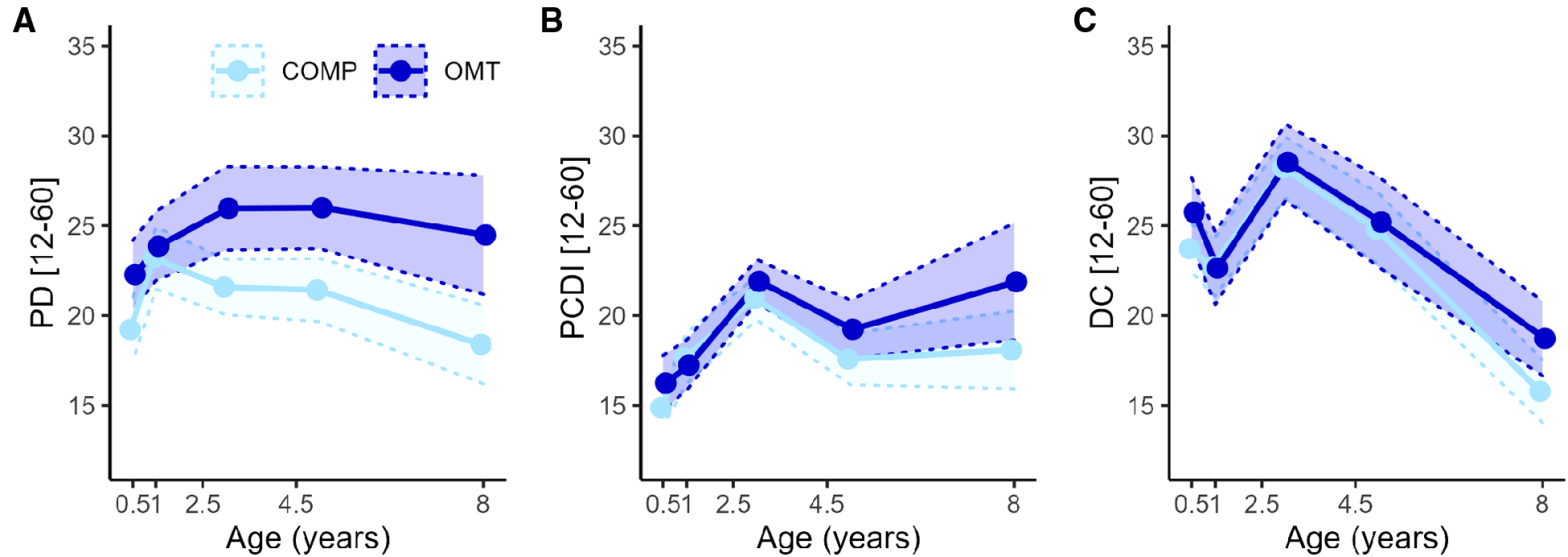
The lines with points in figures **A–C** show the average PSI-SF score across the five assessment points for the three subscales of the PSI-SF. (**A**) Parental distress, (**B**) Parent Child Dysfunctional Interaction and (**C**) Difficult Child. The shaded areas within the dotted lines represent the 95% confidence intervals. The *x*-axis is linearly scaled with time to illustrate the measurement intervals.

For the Parenting Distress (PD) subscale there was a significant main effect of *group* (*F*_1,71.7_ = 9.7, *p* = 0.003) and *age* (*F*_4,244.7_ = 8.7, *p* < 0.0001) and group*age interaction (*F*_4,244.7_ = 3.6, *p* = 0.007). *Post hoc* contrasts showed significant group differences at every assessment apart from 1 year. For the DC sub-scale there was no significant main (*F*_1,71.9_ = 0.8, *p* = 0.38) or interaction effects (*F*_4,246.5_ = 1.4, *p* = 0.25) involving *group*, but a main effect of *age* (*F*_4,246.5_ = 47.4, *p* < 0.0001). For the PCDI subscale there were no significant main (*F*_1,73.6_ = 3.4, *p* = 0.07) or interaction effects (*F*_4,249_ = 2.3, *p* = 0.06) of *group*, but a main effect of *age* (*F*_4,249_ = 23.4, *p* < 0.0001). Figure 3 shows the average scores and confidence intervals on the three subscales which compose the PSI total score across the study period.

### 3.2. Symptoms of psychological distress

#### 3.2.1. Reported depression and anxiety: The hopkins symptom checklist (SCL-25)

**Figure 4:**
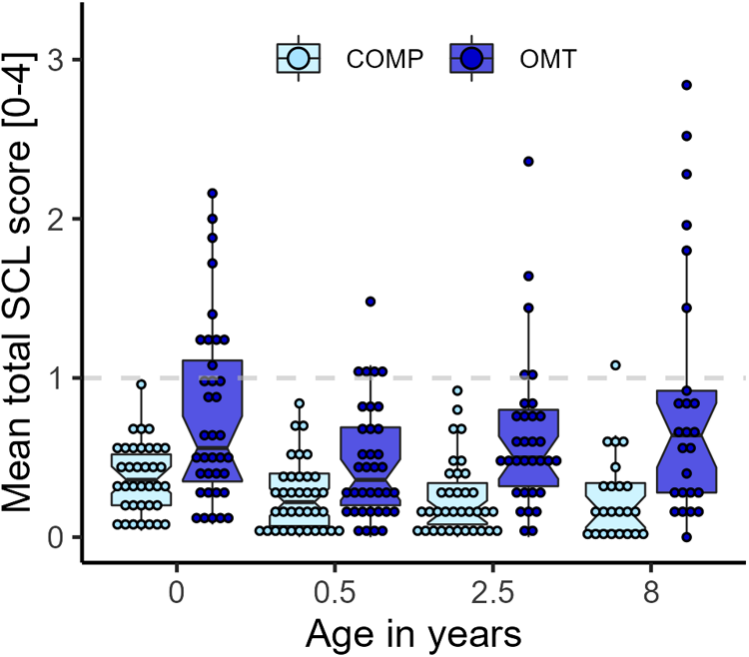
Overview of the average SCL-25 scores by group and measurement occasion. Dots display average score per participant at each assessment. SCL-25 ratings from 4 ½ years were not available.

The SCL-25 was administered in the last trimester of pregnancy, when the child was 6 months, 2.5 years, 4.5 and 8 years. Figure 4 shows that the average SCL-25 score was higher in the OMT group across the whole study period from pregnancy (age = 0) to 8 years (main effect: *F*_1,70_ = 15.6, *p* = 0.0002) with a large spread of scores. Group contrasts showed that there were significant differences at each measurement point (ranging between 5 and 13 points difference in sum score, all *p*'s < 0.0046). Seven women in the OMT group scored ≥1 on average across all measurements. There was a significant main effect of age (*F*_3,181_ = 15.6, *p* < 0.0001), and a group*age interaction effect for the total SCL-25 score (*F*_3,181_ = 3.17, *p* = 0.026). Separate models for anxiety and depression subscales showed similar and robust group differences across the measurements (*p*'s < 0.0006).

The sensitivity analysis of baseline scores for the participants who completed all assessments and those who discontinued at some point during the study, showed no significant difference in SCL-25 score for either group [OMT: mean difference (*Δ*) = 0.06, *p* = 0.77; COMP: *Δ* = −0.01, *p* = 0.91]. Therefore, it is not evident that those who discontinued or were excluded from the study had worse mental health than those who did not.

#### 3.2.2. The Edinburgh postnatal depression scale (EPDS)

**Figure 5:**
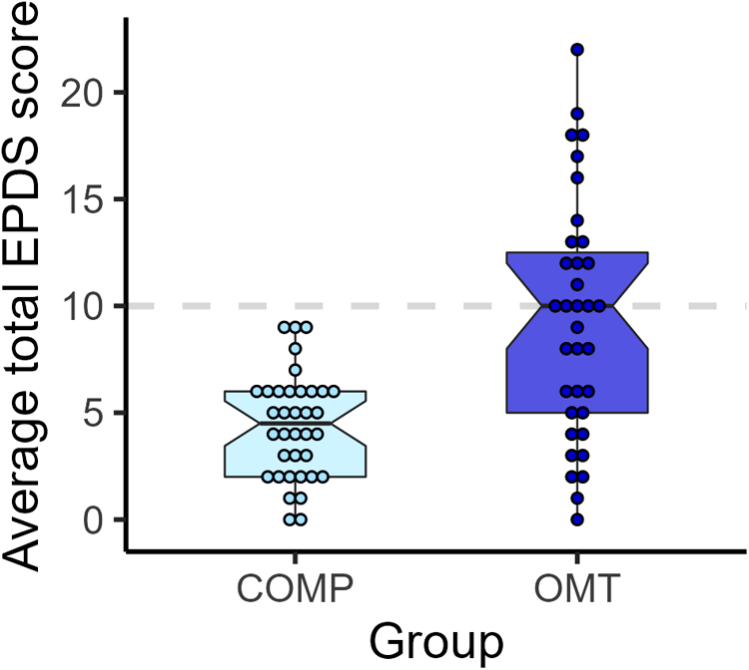
Boxplots and dots showing individual EPDS sum scores for each participant in the two study groups.

We observed large differences in the average scores on the EPDS for the two groups (see Figure 5. Welch's *t*_46.3_ = 4.8, *p* < 0.0001, Cohen's *d* = 1.1), but also a large difference in the spread of scores. While the average of the OMT group was just barely below the cut-off for maternal post-partum depression (scores > 10) on the EPDS, none of the participants in the comparison group scored above this cutoff 3 months after delivery (M ± SD: COMP = 4.49 ± 2.44, OMT = 9.34 ± 5.58).

##### 3.2.2.1. Associations between EPDS and parental distress

There was a moderate significant correlation between the EPDS score and the total PSI-SF score at 6 months (*r* = 0.24, *p* = 0.040) and the EPDS score and the parental distress (PD) subscale at 6 months (*r* = 0.29, *p* = 0.013). Group-wise analyses showed that this association was solely due to a significant correlation in the comparison group (COMP: *r* = 0.4.0, *p* = 0.016; OMT: *r* = 0.11, *p* = 0.5).

### 3.3. Parent reports of child problem behavior

**Figure 6:**
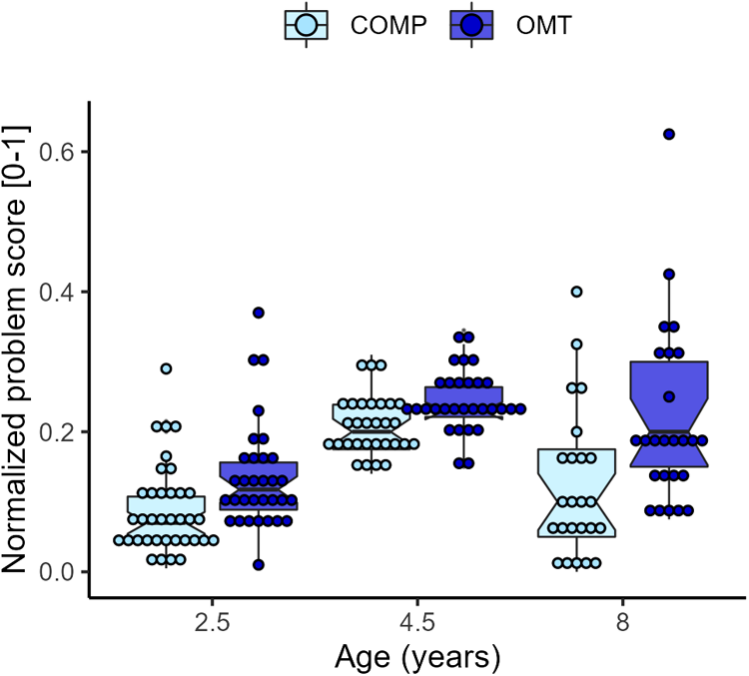
Boxplots of the average rescaled (0–1) problem score per group and age. Individual averages are superimposed as dots on the boxplots.

Figure 6 shows the distribution of parent–reported problem scores collected when the children were 2.5, 4.5 and 8 years old. The mixed model of the (normalized) behavioral problem scores showed a significant effect of *group* (*F*_1,70.5_ = 15.0, *p* = <0.001) and *age* (*F*_2,119.4_ = 52.3, *p* < 0.001), and a significant interaction effect (*F*_2,119.4_ = 3.8, *p* = 0.024). The problem reports were on average 7% higher in the OMT group. In both groups, average scores were higher at the 4.5-year assessment. While reported difficulties significantly decreased from 4.5 to 8 years in the comparison group (*t*_105_ = 4.3, *p* < 0.001), scores in the OMT did not (*t*_105_ = 1.18, *p* = 0.47, Tukey adjustment). The variability in scores was notably higher in both groups at the 8-year assessment (see Figure 6). The ICC was 0.38. A separate regression analysis in the OMT data, showed that neonatal abstinence syndrome (NAS) was not a significant predictor of behavior problems, and did not improve the model fit. Two sensitivity analyses showed group differences in the raw problem score data (CBCL and SDQ), results and descriptive statistics can be found in the Supplementary Material.

#### 3.3.1. Associations between parenting stress and child behavior problems

Pearson correlations between total parenting stress and child behavior problems at the three assessment points: 2.5, 4.5 and 8 years are displayed in Table 3. Coefficients are shown separately for the two groups. Overall, there were medium and strong correlations between scores across time within both outcome measures in the two groups. There was strong association between total PSI score and child behavior problems both at child ages 4.5 (*r* = 0.74, *p* < 0.0001) and 8 (*r* = 0.72, *p* < 0.0001) in the OMT data. Similarly, for the comparison group the correlation between total PSI score and child behavior problems was highest at 4.5 (*r* = 0.68, *p* < 0.0001) and 8 years (*r* = 0.63, *p* = 0.0014). Because the group differences were noticeably more robust on the PD (parental distress) subscale, we also tested the correlation between the PD and SDQ reports at 8 years which showed a significant positive association (*t*_45_ = 4.5, *p* < 0.0001).

**Table 3:** Pearson correlations for main outcomes within the two study groups.

	OMT group					
Prob 2	Prob 4	Prob 8	PSI_tot 2	PSI_tot 4	PSI_tot 8
Comparison group	Prob 2		***0*** ***.*** ***74***	***0*** ***.*** ***56***	**0** **.** **53**	***0*** ***.*** ***57***	**0** **.** **47**
	Prob 4	**0** **.** **45**		***0*** ***.*** ***69***	***0*** ***.*** ***60***	***0*** ***.*** ***74***	***0*** ***.*** ***67***
	Prob 8	0.36	0.36		**0** **.** **39**	**0** **.** **50**	***0*** ***.*** ***72***
	PSI_tot 2	**0** **.** **44**	**0** **.** **49**	**0** **.** **52**		***0*** ***.*** ***63***	**0** **.** **46**
	PSI_tot 4	0.26	***0*** ***.*** ***68***	**0** **.** **54**	***0*** ***.*** ***60***		***0*** ***.*** ***68***
	PSI_tot 8	0.29	0.41	***0*** ***.*** ***63***	***0*** ***.*** ***72***	**0** **.** **61**	

The matrix shows the correlations between problem and stress (PSI total) measures at the three measurement points where both outcomes were assessed. Upper right part of the matrix shows correlations for the OMT group the lower left part shows the corresponding correlations for the comparison group [all correlations significant at the unadjusted 0.05 level are marked in bold. Correlations significant after Bonferroni correction 0.05/30 tests (*p* < 0.0017) are marked in *italic*].

## 4. Discussion

This study describes the trajectories of parenting stress, mental health and reports of child behavior problems in a cohort of women in opioid agonist treatment during pregnancy and their children. The mother-child dyads were followed for 8 years alongside a comparison group of mothers without history of substance use. On average, mothers in the OMT group reported poorer mental health, more child behavior problems and more parenting stress and distress throughout the study period. At the same time, group differences were rather small, and few scored above clinical cuff-offs. Indeed, the group differences were largely due to a handful of participants with high scores across outcomes. Altogether, mothers in stable OMT share many of the same challenges of parenthood with mothers from a normative sample.

Although mothers in the OMT group reported somewhat higher parenting stress than mothers in the comparison group, there were large within-group variability in scores (see Figure 2, 2). Both study groups demonstrated similar patterns in parenting stress over time: increasing levels of stress towards toddlerhood and decreasing stress as children grew older. However, the distribution of scores at the subscales level of the PSI differed in the two groups. While there were no significant group differences in parent-child dysfunctional interaction (PCDI) or perceptions of child difficulties (DC), we found a significant and robust difference in reported parental distress (PD) from toddlerhood onwards. Further inspection of the longitudinal trajectory of parenting stress showed a peak in both groups when the children were 2 ½ years old. This finding is consistent with studies showing that parents experience higher stress in toddlerhood that typically decreases with increasing child age (26, 52). Considering that most 2- and 3-year-olds have tantrums, can resist parental direction and say no to many things, toddlerhood is challenging for most parents. The resistance and protesting behavior that typically characterizes toddlerhood would likely increase the demands of parenting and exacerbate parenting stress. In addition, whether the child is able to successfully regulate emotions is important because it is implicated in behaviors which are characteristics of externalizing behavior problems (53, 54).

It is unsurprising that mothers with opioid addiction have more stress in their lives compared to a low-risk group of mothers without a history of addiction. Individuals in established OMT typically have a more stable lifestyle than individuals with opioid addiction outside treatment (55), but more psychosocial and psychiatric vulnerability than healthy comparison groups (56). Sociodemographic risk factors act as distal stressors in both addicted and non-addicted mothers, which likely reduces tolerance for subjective stress experienced in the parenting role (26, 57). Other distal sources of stress for mothers in OMT may be associated with aspects of the treatment itself. We suggest that the types of surveillance mothers are subjected to in the national OMT of this country acts for better and for worse: On the one hand, the guidelines require close monitoring of women and children, especially during pregnancy and the first year after birth. This may cause stress and fear of making mistakes (41) in mothers who have had histories with child welfare involvement or have experienced removal of children earlier. On the other hand, a coordinated treatment program facilitates access to many services that can help mothers to cope with parenting challenges that arise. Observation, guidance and parental training services are offered both as residential treatment and home-based assistance. Seven women in this study stayed in a mother-infant facility before and after delivery for shorter or longer time, and more than half the women in the OMT group received support from Child Welfare Services (CWS). In a previous paper based on the same cohort, it was reported that CWS had been involved in 19 out of 26 families when the children were 4 years old (58) primarily by offering assistance such as daycare, visiting homes and parental counseling, but also with out-of-home placements of the children. At the last assessment point in this study, seven of the original 36 children in this study had been moved into foster care.

Despite moderate differences in total parenting stress over time, there were group differences in reported anxiety and depression symptoms (SCL-25) that persisted throughout the study. These were largely driven by a subgroup of mothers with high symptom load (Figure 4). Seven mothers in the OMT group scored higher than the cutoff (>1) on depression symptoms and four on the anxiety subscale of the SCL-25. Further, nine of the 36 mothers in the OMT group scored above the clinical cut-off for postnatal depression when their children were 3 months old as measured by the EPDS. At the same time, none of the mothers in the comparison group scored in the clinical range for postnatal depression. A risk factor commonly associated with maternal addiction is psychological maladjustment especially increased symptoms of anxiety and depression (59). These conditions are comorbid and associated with adverse child outcomes (60).

Parental distress was significantly correlated with postnatal depressive symptoms—but only in the comparison group. Both these measures may reflect more general life circumstances and psychosocial burden, not specifically related to the parenting role as such. For example, many of the mothers in the OMT group lived in residential care prior to and after delivery. While the stay at an institution entails a lot of care, verbal reports indicate that these women experience considerable worries about potential relapse and consequently losing custody of their children. It is also likely that the prevalence of neonatal abstinence symptoms, causing worries or difficulties with stress coping. Interestingly, in a previous paper on the same women it was found that while depressive symptoms were significantly reduced from the last month of pregnancy to 6 months later, the trend reversed from 6 months after birth to 2 years later (38).

Reports of child behavior problems were significantly higher in the OMT group at all three points of assessment. In both groups, the highest level of behavior problems was reported at 4.5 years. However, while behavior problems decreased between 4.5 and 8 years in the comparison group, they remained higher and relatively stable in the OMT group in the same period. This result may be a sign of *de facto* more behavior problems among opioid-exposed children compared to non-exposed peers. Alternatively, mothers in OMT who struggle more with the parental role (high PD scores) may also perceive children as “difficult”. According to a transactional model of development, there are dynamic, reciprocal processes of continuous interaction between a child and the caregiving environment (61). It was previously found that parents report somewhat more behavior problems than teachers when the children were 8 years old, but the overall interrater agreement was high (41).

There was a strong positive correlation between parenting stress and child behavior problems among mothers in OMT and a somewhat weaker association in the comparison group. These data align well with previous studies (62, 63) showing that parenting stress was positively associated with behavior problems from infancy to childhood (64). Future studies with larger sample sizes are needed to study the causal relationships between child behavior problems and parenting stress over time and include relevant mediating and moderating factors into the models. Variability in behavior problem scores was notably higher in both groups at 8 years. Also, previous studies showed increasing variation with increasing age (65, 66).

The findings presented here have implications for clinical practice and future research. First, the continuous heightened rates of psychological distress symptoms that characterize mothers in OMT should be given attention from the addiction treatment field and is also relevant for child mental health services. Importantly, the large spread in scores indicates notable individual differences that need to be addressed in terms of differentiated services and measures tailored to each family's individual needs. The bidirectional nature of parenting stress and child behavior problems necessitates a keen-eyed perspective on this complexity.

The SDQ and CBCL are among the most commonly used screening methods for assessing the presence of potential behavior problems in children. Although these instruments provide descriptions of a child's behavior, clinical use is often pragmatic and context dependent: Whilst a cut-off score on symptom-loaded questionnaires may differentiate between children needing intervention and those who do not, the clinical decisions largely depend on a collection of additional information. Notably, scores above or below the clinical thresholds do not always correspond to the parent's own perception of the significance of the child's behavior problems.

One key finding here is that although overall parenting stress was higher in the OMT group, the differences were largely explained by higher parental distress ratings from toddlerhood onwards. It is suggested here that parental distress in this vulnerable group of mothers-child dyads may be a key factor for clinicians when planning counseling, guidance and supportive measures.

### 4.1. Strengths and limitations

The longitudinal design of the study is a key strength of the study. A selection of outcomes related to demographics, life circumstances, psychosocial development, health and well-being were collected over almost a decade. As such, the OMT group who provided data for the present study is unique in a national and international context Compared to many other studies of outcomes related to OMT, this group led a relatively stable lifestyle with fewer psychosocial vulnerability factors. Also, most of the women in this study maintained custody of their children. More than two-thirds of the women recruited nationally completed the whole study and retention rate was highest in the OMT group. Mothers in current OMT knew that their responses would not be shared with treatment providers or CWS. The result was an excellent working alliance with the participants as reflected in the high retention rate. Further, the OMT group were subjected to very tight follow-up during the first years of the study period and had minimal on-top use of illicit drugs (38, 56).

Several limitations should be acknowledged. Firstly, the small sample size from a single country limits the external validity of the study and the generalizability of the results. However, all women who gave birth while in OMT program in Norway during the inclusion interval were invited to participate, and 76% did. Our study has limited statistical power with 24–36 datapoints per assessment. However, the correlations between within-subject measurements across time was high (ICC) and statistical models that can accommodate all the available data were used. However, the modest number of participants also limited the choices of statistical approach. More advanced methods (such as structural equation models) would allow assessment of the directional effects of the main outcomes measured at multiple timepoints but require larger study samples. Future studies are needed to assess potential causal relationships between parenting stress, child behavioral problems and mental health.

Because there was some loss of data over time, the results from the latest timepoints should be interpreted with some caution as missing data may not be unrelated to the main outcomes. To assess this, a sensitivity analysis was conducted to check whether the parents that were excluded or discontinued the study had worse mental health at the first assessment (in pregnancy) compared to those that completed the whole study. This analysis showed no difference in mental health at study entry between those who completed the study and those who discontinued. Further, the PSI data analyzed here come from two different versions, with overlapping items. While using the same version of the PSI would have been ideal, the Cronbach alpha (*α*) showed satisfactory consistency.

Here parental stress, mental health and child behavior problems were based solely on self-reports from the same individuals (mothers). Using several data sources can diminish the effects of reporting bias. However, in a recent paper findings showed high agreement between parents and teachers on reported SDQ at child age 8 years in this cohort (41).

A key limitation to consider is that the “normative” comparison group was only matched on age and time of pregnancy. There were considerable differences in sociodemographic factors and life circumstances in the two groups. For instance, nearly all mothers in the OMT group and none in the comparison group were smokers. This is in line with previous research showing that 97% of pregnant women in opioid maintenance treatment used tobacco (67). Studies have suggested that prenatal exposure to tobacco is associated with attentional deficits, behavioral problems, as well as impaired memory function (68). Children born to opioid-maintained women may therefore be potentially at double risk for negative developmental outcomes from both prenatal opioid and nicotine exposure. Altogether, this could mean that the group differences found here may be inflated and could be smaller if the comparison group had been matched on smoking, sociodemographic variables, and other risk factors. It is noteworthy that despite the high rate of smoking among women in the maintenance treatment, group differences remained small. This may indicate that smoking has less effect on child behavior over time when other maternal lifestyle factor are under control.

The families in this study have been followed by the same two researchers over nearly a decade. This has undoubtedly led to high retention in the study but may also have had some influence on the outcomes of this study. At each timepoint, most participants consented to be contacted again, and expected invitation to follow-up assessments. This may by itself have conferred support and constituted a stabilizing factor in life of individuals who have experienced a lot of turbulence with treatment providers and authorities. Also, the study's explicit aim to study developmental trajectories in children could prompt caregiving competency in parents who have many concerns about their children and the potential harm caused by prenatal opioid exposure.

These data may also be relevant in light of the stark increase in prescription opioid misuse in many countries. The relatively stable group tested here with a history of heroin addiction and long-term OMT may share characteristics with patients who develop opioid addiction following pain treatment, and the results may therefore better generalize to these patients than individuals with current illicit drug use. The OMT group tested here present with higher problem load and stress than the community sample, yet most individuals have scores that fall within the non-clinical range. It is also possible that the mothers in this OMT group is exposed to fewer and different life stressors than most women currently using drugs illicitly. Accordingly, high stress and problem behavior is not a necessary, direct consequence of prenatal exposure to opioids.

### 4.2. Conclusion

Increasing use and misuse of opioids can have ripple effects on families. In pregnancy, opioid addiction is recognized as a major risk factor, and there is concern for the growing number of children exposed to opioids prenatally and potential adverse developmental outcomes. While findings showed somewhat higher parental distress and child behavior problems in the OMT group assessed here, scores are largely in the sub-clinical range across a time period of 8 years. Compared to other study samples, mothers in this group had little problems with illicit drug use and provided a stable caregiving environment for their children over time. Consequently, it is suggested that prenatal opioid exposure by itself does not cause developmental problems. This notwithstanding, a small number of the dyads studied here scored in the clinical range across several measures which underscore the need to identify high-risk mother-child dyads in order to render specialized services and tailored follow-up measures.

## Data Availability

The data analyzed in this study is not publicly accessible due to privacy constraints. The R code for the analyses presented here is available from the authors on request. Requests to access these datasets should be directed to marie.eikemo@psykologi.uio.no.

## References

[1] Crowley DM Connell CM Jones D Donovan MW. Considering the child welfare system burden from opioid misuse: research priorities for estimating public costs. Am J Manag Care. (2019) 25(13 Suppl):S256. PMID: ; PMCID: 31361428 PMC7895335

[2] Guo C Moses-Kolko E Phillips M Swain JE Hipwell AE. Severity of anxiety moderates the association between neural circuits and maternal behaviors in the postpartum period. Cogn Affect Behav Neurosci. (2018) 18(3):426–36. 10.3758/s13415-017-0516-x 29619759 PMC6546103

[3] Whittaker A. Guidelines for the identification and management of substance use and substance use disorders in pregnancy. Geneva, Switzerland: WHO Press (2014). 224 p. 24783312

[4] Mozurkewich EL Rayburn WF. Buprenorphine and methadone for opioid addiction during pregnancy. Obstet Gynecol Clin North Am. (2014) 41(2):241–53. 10.1016/j.ogc.2014.02.005 24845488

[5] Jansson LM Di Pietro JA Elko A Williams EL Milio L Velez M. Pregnancies exposed to methadone, methadone and other illicit substances, and poly-drugs without methadone: a comparison of fetal neurobehaviors and infant outcomes. Drug Alcohol Depend. (2012) 122(3):213–9. 10.1016/j.drugalcdep.2011.10.003 22041255 PMC3288292

[6] Jones HE O'Grady KE Malfi D Tuten M. Methadone maintenance vs. methadone taper during pregnancy: maternal and neonatal outcomes. Am J Addict. (2008) 17(5):372–86. 10.1080/10550490802266276 18770079

[7] Muller AE Skurtveit S Clausen T. Building abstinent networks is an important resource in improving quality of life. Drug Alcohol Depend. (2017) 180:431–8. 10.1016/j.drugalcdep.2017.09.006 28988006

[8] Conradt E Crowell SE Lester BM. Early life stress and environmental influences on the neurodevelopment of children with prenatal opioid exposure. Neurobiol Stress. (2018) 9:48–54. 10.1016/j.ynstr.2018.08.005 30151420 PMC6108075

[9] Monnelly VJ Hamilton R Chappell FM Mactier H Boardman JP. Childhood neurodevelopment after prescription of maintenance methadone for opioid dependency in pregnancy: a systematic review and meta-analysis. Dev Med Child Neurol. (2019) 61(7):750–60. 10.1111/dmcn.14117 30511742 PMC6617808

[10] Kaltenbach K O’Grady KE Heil SH Salisbury AL Coyle MG Fischer G Prenatal exposure to methadone or buprenorphine: early childhood developmental outcomes. Drug Alcohol Depend. (2018) 185:40–9. 10.1016/j.drugalcdep.2017.11.030 29413437 PMC5906792

[11] Arter SJ Tyler B McAllister J Kiel E Güler A Cameron Hay M. Longitudinal outcomes of children exposed to opioids in-utero: a systematic review. J Nurs Scholarsh. (2021) 53(1):55–64. 10.1111/jnu.12609 33225521

[12] Baldacchino A Arbuckle K Petrie D McCowan C. Neurobehavioral consequences of chronic intrauterine opioid exposure in infants and preschool children: a systematic review and meta-analysis. BMC Psychiatry. (2014) 14(1):1–12. 10.1186/1471-244X-14-104 PMC4021271 24708875

[13] Azuine R Ji Y Chang H-Y Kim Y Ji H DiBari J Prenatal risk factors and perinatal and postnatal outcomes associated with maternal opioid exposure in an urban, low-income, multiethnic US population. JAMA Network Open. (2019) 2:e196405. 10.1001/jamanetworkopen.2019.6405 31251378 PMC6604084

[14] Bakstad B Welle-Strand GK. Nasjonal retningslinje for gravide i legemiddelassistert rehabilitering (LAR) og oppfølging av familiene frem til barnet når skolealder [Only in Norwegian: National guidelines for pregnant women in opioid maintenance treatment (OMT) and follow-up of their families until the children reach school age]. Oslo, Norway: Norwegian Directorate of Health (2011).

[15] Lund IO Skurtveit S Sarfi M Bakstad B Welle-Strand G Ravndal E. Substance use during and after pregnancy among a national cohort of pregnant women in opioid maintenance treatment and their partners. J Subst Use. (2012) 17(3):277–86. 10.3109/14659891.2011.580415

[16] Welle-Strand GK Skurtveit S Jones HE Waal H Bakstad B Bjarkø L Neonatal outcomes following in utero exposure to methadone or buprenorphine: a national cohort study of opioid-agonist treatment of pregnant women in Norway from 1996 to 2009. Drug Alcohol Depend. (2013) 127(1):200–6. 10.1016/j.drugalcdep.2012.07.001 22841456

[17] Sarfi M Eikemo M Welle-Strand GK Muller AE Lehmann S. Mental health and use of health care services in opioid-exposed school-aged children compared to foster children. Eur Child Adolesc Psych. (2022) 31(3):495–509. 10.1007/s00787-021-01728-3 PMC8940845 33590310

[18] Peisch V Sullivan A D Breslend NL Benoit R Sigmon SC Forehand GL Parental opioid abuse: a review of child outcomes, parenting, and parenting interventions. J Child Fam Stud. (2018) 27(7):2082–99. 10.1007/s10826-018-1061-0

[19] Koob GF Volkow ND. Neurobiology of addiction: a neurocircuitry analysis. Lancet Psychiatry. (2016) 3(8):760–73. 10.1016/S2215-0366(16)00104-8 27475769 PMC6135092

[20] Rutherford HJV Kim S Yip SW Potenza MN Mayes LC Strathearn L. Parenting and addictions: current insights from human neuroscience. Curr Addict Rep. (2021) 8(3):380–8. 10.1007/s40429-021-00384-6 36185758 PMC9523670

[21] Swain JE Ho SS. Early postpartum resting-state functional connectivity for mothers receiving buprenorphine treatment for opioid use disorder: a pilot study. J Neuroendocrinol. (2019) 31(9):e12770. 10.1111/jne.12770 31287922 PMC7195812

[22] Crnic KA Greenberg MT. Minor parenting stresses with young children. Child Dev. (1990) 61(5):1628–37. 10.2307/1130770 2245752

[23] Hakvoort EM Bos HMW Van Balen F Hermanns JMA. Spillover between mothers’ postdivorce relationships: the mediating role of parenting stress. Pers Relatsh. (2012) 19(2):247–54. 10.1111/j.1475-6811.2011.01351.x

[24] Killeen TCS Brady KT. Parental stress and child behavioral outcomes following substance abuse residential treatment: follow-up at 6 and 12 months. J Subst Abuse Treat. (2000) 19(1):23–9. 10.1016/S0740-5472(99)00078-1 10867297

[25] Rutherford HJV Mayes LC. Parenting stress: a novel mechanism of addiction vulnerability. Neurobiol Stress. (2019) 11:100172. 10.1016/j.ynstr.2019.100172 31193862 PMC6543178

[26] Mackler JS Kelleher RT Shanahan L Calkins SD Keane SP O'Brien M. Parenting stress, parental reactions, and externalizing behavior from ages 4 to 10. J Marriage Fam. (2015) 77(2):388–406. 10.1111/jomf.12163 26778852 PMC4712732

[27] Kelley SJ. Stress and coping behaviors of substance-abusing mothers. J Spec Pediatr Nurs. (1998) 3(3):103. 10.1111/j.1744-6155.1998.tb00215.x 9743924

[28] Kelley SJ. Parenting stress and child maltreatment in drug-exposed children. Child Abuse Negl. (1992) 16(3):317–28. 10.1016/0145-2134(92)90042-P 1617467

[29] Hatzis D Dawe S Harnett P Barlow J. Quality of caregiving in mothers with illicit substance use: a systematic review and meta-analysis. Subst Abus. (2017) 11:1178221817694038. 10.1177/1178221817694038 PMC5398331 28469425

[30] Velez M Jansson L. The opioid dependent mother and newborn dyad: nonpharmacologic care. J Addict Med. (2008) 2(3):113–20. 10.1097/ADM.0b013e31817e6105 19727440 PMC2729936

[31] Abidin RR. The determinants of parenting behavior. J Clin Child Psychol. (1992) 21(4):407–12. 10.1207/s15374424jccp2104_12

[32] Holly LE Fenley AR Kritikos TK Merson RA Abidin RR Langer DA. Evidence-base update for parenting stress measures in clinical samples. J Clin Child Adolesc Psychol. (2019) 48(5):685–705. 10.1080/15374416.2019.1639515 31393178

[33] Wouldes TA Woodward LJ. Maternal methadone dose during pregnancy and infant clinical outcome. Neurotoxicol Teratol. (2010) 32(3):406–13. 10.1016/j.ntt.2010.01.007 20102736

[34] Odsbu I Mahic M Skurtveit SO Lund I-O Handal M. An 11-year nationwide registry-linkage study of opioid maintenance treatment in pregnancy in Norway. Norsk Epidemiologi. (2021) 29(1-2):63–70. 10.5324/nje.v29i1-2.4047

[35] Bakstad B Sarfi MA Welle-Strand GK Ravndal E. Opioid maintenance treatment during pregnancy: occurrence and severity of neonatal abstinence syndrome. Eur Addict Res. (2009) 15(3):128–34. 10.1159/000210042 19332991

[36] Sarfi M Smith L Waal H Sundet JM. Risks and realities: dyadic interaction between 6-month-old infants and their mothers in opioid maintenance treatment. Infant Behav Dev. (2011) 34(4):578–89. 10.1016/j.infbeh.2011.06.006 21824659

[37] Sarfi M. Vulnerable but adaptable. A longitudinal study of a national cohort of children born to women in opiod maintenance (OMT) treatment. [dissertation]. Oslo: University of Oslo (2012).

[38] Lund IO Skurtveit S Sarfi AM Bakstad B Welle-Strand G Ravndal E. A 2-year prospective study of psychological distress in pregnant women in opioid maintenance treatment and their partners. J Subst Use. (2013) 18(2):148–60. 10.3109/14659891.2011.642928

[39] Konijnenberg C Lund IO Melinder A. Behavioural outcomes of four-year-old children prenatally exposed to methadone or buprenorphine: a test of three risk models. Early Child Dev Care. (2015) 185(10):1641–57. 10.1080/03004430.2015.1016506

[40] Welle-Strand GK Skurtveit S Abel KF Chalabianloo F Sarfi M. Living a normal life? Follow-up study of women who had been in opioid maintenance treatment during pregnancy. J Subst Abuse Treat. (2020) 113:108004. 10.1016/j.jsat.2020.108004 32359675

[41] Abidin RR. Parenting stress index-short form. Charlottesville, VA: Pediatric Psychology Press (1990).

[42] Abidin RR. Parenting stress index: Manual. 3rd ed. Odessa, FL: Psychological Assessment Resources, Inc. (1995).

[43] Abidin RR. Parenting stress index-fourth edition (PSI-4). Lutz, FL: Psychological Assessment Resources (2012).

[44] Haskett ME Ahern LS Ward CS Allaire JC. Factor structure and validity of the parenting stress index-short form. J Clin Child Adolesc Psychol. (2006) 35(2):302–12. 10.1207/s15374424jccp3502_14 16597226

[45] Cox JL Holden JM Sagovsky R. Detection of postnatal depression: development of the 10-item Edinburgh postnatal depression scale. Br J Psychiatry. (1987) 150(6):782–6. 10.1192/bjp.150.6.782 3651732

[46] Levis B Negeri Z Sun Y Benedetti A Thombs BD. Accuracy of the Edinburgh postnatal depression scale (EPDS) for screening to detect major depression among pregnant and postpartum women: systematic review and meta-analysis of individual participant data. Br Med J. (2020) 371:m4022. 10.1136/bmj.m4022 33177069 PMC7656313

[47] Derogatis LR Lipman RS Rickels K Uhlenhuth EH Covi L. The hopkins symptom checklist (HSCL): a self-report symptom inventory. Behav Sci. (1974) 19(1):1–15. 10.1002/bs.3830190102 4808738

[48] Lund IO. Pregnant women in opioid maintenance treatment (OMT): maternal and neonatal outcomes. Oslo: University of Oslo (2013).

[49] Achenbach TM. Manual for the child behavior checklist/2-3. Burlington, VT: University of Vermont, Department of Psychiatry (1992).

[50] Lavigne JV Meyers KM Feldman M. Systematic review: classification accuracy of behavioral screening measures for use in integrated primary care settings. J Pediatr Psychol. (2016) 41(10):1091–109. 10.1093/jpepsy/jsw049 27289069

[51] Sheldrick RC Benneyan JC Kiss IG Briggs-Gowan MJ Copeland W Carter AS. Thresholds and accuracy in screening tools for early detection of psychopathology. J Child Psychol Psychiatry. (2015) 56(9):936–48. 10.1111/jcpp.12442 26096036 PMC4532658

[52] Williford AP Calkins SD Keane SP. Predicting change in parenting stress across early childhood: child and maternal factors. J Abnorm Child Psychol. (2007) 35(2):251–63. 10.1007/s10802-006-9082-3 17186365

[53] Calkins SD Gill KL Johnson MC Smith CL. Emotional reactivity and emotional regulation strategies as predictors of social behavior with peers during toddlerhood. Soc Dev. (1999) 8(3):310–34. 10.1111/1467-9507.00098

[54] Calkins SD Dedmon SE. Physiological and behavioral regulation in two-year-old children with aggressive/destructive behavior problems. J Abnorm Child Psychol. (2000) 28(2):103–18. 10.1023/A:1005112912906 10834764

[55] Skinner ML Haggerty KP Fleming CB Catalano RF Gainey RR. Opiate-addicted parents in methadone treatment: long-term recovery, health, and family relationships. J Addict Dis. (2010) 30(1):17–26. 10.1080/10550887.2010.531670 PMC3025601 21218307

[56] Sarfi M Sundet JM Waal H. Maternal stress and behavioral adaptation in methadone- or buprenorphine-exposed toddlers. Infant Behav Dev. (2013) 36(4):707–16. 10.1016/j.infbeh.2013.08.006 23999378

[57] Suchman N Luthar SS. The mediating role of parenting stress in methadone-maintained mothers’ parenting. Parenting. (2001) 1(4):285–315. 10.1207/S15327922PAR0104_2 17710214 PMC1949392

[58] Lund IO Brendryen H Ravndal E. A longitudinal study on substance use and related problems in women in opioid maintenance treatment from pregnancy to four years after giving birth. J. Subst. Abuse Treat. (2014) 8:35–40. 10.4137/SART.S15055 PMC4024055 24855370

[59] Seay KD Kohl PL. The comorbid and individual impacts of maternal depression and substance dependence on parenting and child behavior problems. J Fam Violence. (2015) 30(7):899–910. 10.1007/s10896-015-9721-y 26478656 PMC4607289

[60] Barker ED Jaffee SR Uher R Maughan B. The contribution of prenatal and postnatal maternal anxiety and depression to child maladjustment. Depress Anxiety. (2011) 28(8):696–702. 10.1002/da.20856 21769997

[61] Sameroff AJ. The transactional model. In: Sameroff AJ, editors. The transactional model of development how children and contexts shape each other. 1st ed. Washington, DC: American Psychological Association (2009). p. 3–21.

[62] Benzies KM Harrison MJ Magill-Evans J. Parenting stress, marital quality, and child behavior problems at age 7 years. Public Health Nurs. (2004) 21(2):111–21. 10.1111/j.0737-1209.2004.021204.x 14987210

[63] Stone LL Mares SHW Otten R Engels RCME Janssens JMAM. The co-development of parenting stress and childhood internalizing and externalizing problems. J Psychopathol Behav Assess. (2016) 38(1):76–86. 10.1007/s10862-015-9500-3 27069304 PMC4789299

[64] Bayer JK Hiscock H Ukoumunne OC Price A Wake M. Early childhood aetiology of mental health problems: a longitudinal population-based study. J Child Psychol Psychiatry. (2008) 49(11):1166–74. 10.1111/j.1469-7610.2008.01943.x 18665879

[65] Neece CL Green SA Baker BL. Parenting stress and child behavior problems: a transactional relationship across time. Am J Intellect Dev Disabil. (2012) 117(1):48–66. 10.1352/1944-7558-117.1.48 22264112 PMC4861150

[66] Newton EK Laible D Carlo G Steele JS McGinley M. Do sensitive parents foster kind children, or vice versa? Bidirectional influences between children’s prosocial behavior and parental sensitivity. Dev Psychol. (2014) 50(6):1808–16. 10.1037/a0036495 24708456

[67] Jones HE Heil SH Tuten M Chisolm MS Foster JM O’Grady KE Cigarette smoking in opioid-dependent pregnant women: neonatal and maternal outcomes. Drug Alcohol Depend. (2013) 131(3):271–7. 10.1016/j.drugalcdep.2012.11.019 23279924 PMC3694998

[68] Zhou S Rosenthal DG Sherman S Zelikoff J Gordon T Weitzman M. Physical, behavioral, and cognitive effects of prenatal tobacco and postnatal secondhand smoke exposure. Curr Probl Pediatr Adolesc Health Care. (2014) 44(8):219–41. 10.1016/j.cppeds.2014.03.007 25106748 PMC6876620

