# Supplementary material for: Children born to women in opioid maintenance treatment: A longitudinal study of child behavioral problems and parenting stress
Source: Front Pediatr. 2022 Dec 23;10:1087956. doi: 10.3389/fped.2022.1087956 (PMC9816796; doi:10.3389/fped.2022.1087956)
Supplement: Supplementary file 1 [file Table1.docx]

Children born to women in OMT: A longitudinal study of parenting stress and child problem behavior

Monica Sarfi^1*^, Marie Eikemo^2*^, Carolien Konijnenberg^3^

1 Norwegian Centre for Addiction Research (SERAF), Faculty of Medicine, University of Oslo, Norway

2 Department of Psychology, Inland Norway University of Applied Sciences, Lillehammer, Norway

3 Department of Psychology, Faculty of Social Sciences, University of Oslo, Norway

*Equal author contribution

Corresponding author

Monica Sarfi

Email: a.m.sarfi@medisin.uio.no

## Sensitivity analysis

The behavior problem scores were normalized in order to assess the development of problems over time on a common scale. Sensitivity analysis of raw data from the CBCL (mixed model) and SDQ (welch’s t) showed parallel results to the scaled (normalized) results, with significant group differences for each scale (see Supplementary table1).

**Supplementary table 1**

|  | COMP Mean (sd) | OMT Mean (sd) | test |
| --- | --- | --- | --- |
| CBCL 2.5 | 24 (23) | 39 (25) | F_1,66_ = 10.9  *p* = .0015 |
| CBCL 4 | 42 (8) | 48 (9) |  |
| SDQ 8 | 5.9 (4.0) | 8.7(5.1) | t_44.3_ = 2.8, *p* =.008 |

COMP = comparison group, OMT = opioid maintenance treatment.

Statistical software:

All analyses and plots were made in R using the packages, and in large via the *Tidyverse (Wickham et al., 2019)*. *Lme4 (version 1.1.27)* was used for fitting mixed effects models (Bates et al., 2015). *Lmertest* (version 3.1.3) for additional statistical output and *Emmeans* for the estimated means and pairwise comparisons (Length, 2021).

## Supplementary References:

1. Wickham H, Averick M, Bryan J, Chang W, McGowan LD, François R, Grolemund G, Hayes A, Henry L, Hester J, Kuhn M, Pedersen TL, Miller E, Bache SM, Müller K, Ooms J, Robinson D, Seidel DP, Spinu V, Takahashi K, Vaughan D, Wilke C, Woo K, Yutani H (2019). “Welcome to the tidyverse.” Journal of Open Source Software, 4(43), 1686. doi: <10.21105/joss.01686>.
2. Bates D, Mächler M, Bolker B, Walker S (2015). “Fitting Linear Mixed-Effects Models Using lme4.” Journal of Statistical Software, **67**(1), 1–48. doi: [10.18637/jss.v067.i01](https://doi.org/10.18637/jss.v067.i01).
3. Russell V. Lenth (2021). emmeans: Estimated Marginal Means, aka Least-Squares Means. R package version 1.6.1. https://CRAN.R-project.org/package=emmeans
